# Poly(Glycerol) Microparticles as Drug Delivery Vehicle for Biomedical Use

**DOI:** 10.3390/pharmaceutics15020384

**Published:** 2023-01-23

**Authors:** Mehtap Sahiner, Aynur S. Yilmaz, Ramesh S. Ayyala, Nurettin Sahiner

**Affiliations:** 1 Department of Bioengineering, Faculty of Engineering, Canakkale Onsekiz Mart University Terzioglu Campus, Canakkale 17100, Turkey; 2 Department of Chemistry, Faculty of Sciences & Arts, and Nanoscience and Technology Research and Application Center (NANORAC), Canakkale Onsekiz Mart University Terzioglu Campus, Canakkale 17100, Turkey; 3 Department of Ophthalmology, Morsani College of Medicine, University of South Florida Eye Institute, 12901 Bruce B Down Blvd, MDC 21, Tampa, FL 33612, USA; 4 Department of Chemical & Biomedical Engineering, Materials Science and Engineering Program, University of South Florida, Tampa, FL 33620, USA

**Keywords:** glycerol micro particles, quercetin delivery, cell/blood compatibility, antioxidant

## Abstract

Glycerol (Gly) is a well-known, FDA-approved molecule posing three hydroxyl groups. Since Gly is biocompatible, here, it was aimed to prepare poly(Glycerol) (p(Gly)) particles directly for the first time for the delivery of therapeutic agents. Micrometer-sized particles of p(Gly) were successfully synthesized via the micro-emulsion method with an average size of 14.5 ± 5.6 µm. P(Gly) microparticles up to 1.0 g/mL concentrations were found biocompatible with 85 ± 1% cell viability against L929 fibroblasts. Moreover, p(Gly) microparticles were tested for hemocompatibility, and it was found that up to 1.0 mg/mL concentrations the particles were non-hemolytic with 0.4 ± 0.1% hemolysis ratios. In addition, the blood compatibility index values of the prepared p(Gly) particles were found as 95 ± 2%, indicating that these microparticles are both bio- and hemocompatible. Furthermore, Quercetin (QC) flavonoid, which possessed high antioxidant properties, was loaded into p(Gly) microparticles to demonstrate drug-carrying properties of the particles with improved bioavailability, non-toxicity, and high biocompatibility. The results of this study evidently revealed that p(Gly) particles can be directly prepared from a cost-effective and easily accessible glycerol molecule and the prepared particles exhibited good biocompatibility, hemocompatibility, and non-toxicity. Therefore, p(Gly) particles were found as promising vehicles for drug delivery systems in terms of their higher loading and release capability as well as for sustained long term release profiles.

## 1. Introduction

Glycerol (Gly), 1,2,3 propanetriol is versatile molecule and take place parts in intriguing biological functions [1]. Gly also called Glycerin is a transparent, odorless, hydroscopic, viscous, and water miscible molecule with high-boiling point (290 °C) in liquid [2]. Since ancient times, Gly has been produced by heating oils and ash (to produce soap). Gly has a wide range of industrial applications, including in the food industry, because it tastes sweet [3]. Due to its use as a biofuel in the energy sector, it is produced at high rates [4]. Since Gly is the main by-product of the biodiesel industry, it is easy to access, abundant, and inexpensive [5]. It is even used as food for piglet’s diet without any side effects [6]. In addition, due to its biological benefits, it is consumed in foods, e.g., as a sweetener, a solvent, and as an emulsifier [7]. In the food packaging industry, Gly has a critical role in providing a plasticizing effect; it enhances the ductility of biodegradable packaging [8]. The physical property of Gly makes it useful as an additive in the pharmaceutical and cosmetic industries, e.g., as a plasticizer, thickener, emollient, demulcent, humectant, bodying agent, lubricant, etc. [9]. It was even reported that Gly can be used in blend films for antibacterial cotton gauze as due its’ plasticization and hydrophilic properties [10]. Gly can also be applied various skin ailments because of the moisture-controlling features [7]. It was demonstrated that the imiquimod mouse model of psoriasis was improved by Gly and pro-inflammatory cytokine expression was inhibited upon application topically [11]. In the antibacterial biomedical applications, chitosan acetate films were treated with Gly to improve their elasticity [12]. Gly was used to dissolve ibuprofen (drug) to create silicone based wound dressing membranes [4]. Moreover, it has been shown that topical application of materials containing Gly improves the skin properties of patients with diseases characterized by xerosis and impaired epidermal barrier function [13]. Further, Gly is used to preserve the human donor skin allografts by tissue banks for the long-term storage of skin grafts [14]. Gly increases epidermal hydration in skin conditions aggravated by dry and cold environmental conditions, such as winter xerosis [13]. Glycerin-based gel sheet (65%) combined with a hydrophilic polymer that absorbs the wound exudate and releases Gly from the gel provides many benefits for wound healing [15]. Gly is an osmotic dehydrating agent that also affects brain metabolism [16]. A dose of 0.25–2.0 g/kg of Gly can decrease intracranial pressure in a variety of diseases, such as stroke, meningitis, pseudotumor cerebri, central nervous system tumors, and space-occupying lesions [16].

Although advanced nanoparticles have been the object of a substantial number of research, fewer have focused on microparticles. Compared with nanosized systems, microparticles have some advantages, including ease of production and characterization, extended-release properties, and high drug loading capabilities [17]. Microparticles can be used as a biopolymeric carriers for wound healing material through re-epithelialization and fibrosis inhibition [18]. Among the applications of microgels are the repair of heart tissue, the treatment of osteoarthritis, and the regeneration of bone and neuronal tissue engineering [19]. The ability to encapsulate cells capabilities by tuning the ability of micro- hydrogels to achieve controlled release of therapeutics is applicable to the development of cell-based therapies. Therefore, currently, the use of microgels for cell sequencing and the use of micro-hydrogels for drug delivery, cell therapy, and cell array-based systems are subject scrutiny [20].

Quercetin (QC) is a flavanol, bitter-tasting, crystalline natural plant pigment, and has an important place in traditional and botanical medicine with its various bioactive properties [21]. The polyphenolic QC, which belongs to the flavonoid class, is mainly found in onions, blackberries, apples, red grapes, tea and red wine [22]. QC has psychostimulant, anti-allergic, and anti-inflammatory properties, maintaining the stability of white blood cells [21], as well as anticancer, anti-viral, anti-diabetic, cardio protective, immunomodulatory, antihypertensive, and gastrointestinal system protective effects. Most importantly, the activities of QC in reducing the risk of infection and antioxidant effects on reactive oxygen species (ROS) has been reported [23,24,25]. Boots et al. revealed that orally administrated QC reduced the oxidative stress related damage by boosting antioxidant defense mechanism [26]. Ou et al., showed that QC could significantly reduce serum C-reactive protein levels, which is an acute inflammation marker [27]. In another study, daily consumption of QC enriched food decreased total cholesterol, LDL-cholesterol, and plasma glucose levels [28].

In this study, p(Gly) particles were synthesized via micro-emulsion method in a single step for the first time. The characterization of p(Gly) particles was performed using FT-IR, thermal gravimetric analyzer (TGA), scanning electron microscope (SEM) image, and zeta potential measurements. Furthermore, the cell compatibility and blood compatibility of p(Gly) particles were evaluated up to 1.0 mg/mL particle concentrations. QC as an active mod compound was loaded to the p(Gly) particles, and the release study of QC were studied under physiological conditions, in PBS buffer at 37 °C. 

## 2. Materials and Methods

### 2.1. Materials

Glycerol (Gly, 99+% extra pure, Acros Organics, Geel, Belgium) and divinyl sulfone (DVS, >96%, TCI) were used as received in p(QC) particle preparation. Quercetin dihydrate (QC, 99.85% MP Biomedicals) was used as active agent. Folin–Ciocalteau’s phenol reagent (FC, Sigma-Aldrich), gallic acid (GA, 97.5–102.5%, Aldrich), Sodium nitrite (Merck, extra pure), and aluminum chloride (Merck, anhydrous powder sublimed from synthesis) were used for antioxidant testing. 

L929 fibroblast cell line (Mouse C3/An connective tissue), obtained from the SAP Institute (Ankara, Turkey), was used for the cell viability tests. Dulbecco’s Modified Eagle’s Medium, DMEM, 4.5 g/L Glucose, sodium pyruvate, L-Glutamine, 3.7 g/L NaHCO_3_ (Pan Biotech GmbH, Aidenbach, Germany) was used as received. Fetal bovine serum (FBS Premium, heat inactivated), antibiotics (penicillin (10,000 U/mL), streptomycin (10,000 μg/mL), and trypsin/EDTA (trypsin 0.25%, 1 mM EDTA-Na_4_ in HBSS) were purchased from Pan Biotech (GmbH, Germany). Trypan Blue solution (0.5%) was acquired from Biological Industries and thiazolyl blue (MTT) was obtained from BioFroxx (Einhausen, Germany). Dimethyl sulfoxide (DMSO, 99.9%, Carlo-Erba) was used as received.

### 2.2. Synthesis and Characterization of P(Gly) Particles

P(Gly) particles were synthesized via a microemulsion method in 30 mL 0.1 M leci-thin/cyclohexane medium. Briefly, 0.3 mL Glycerol was dissolved in 1 mL of 0.5 M NaOH solution. Then, Gly solution of 0.4 mL was put into 30 mL of 0.1 M lecithin/cyclohexane, while stirring at 1000 rpm. Then, 100, 75, 50, 37.5, and 18.8% mol of DVS was added as a crosslinker agent, separately. After one hour mixing at room temperature, the microparticles were participated via centrifuge at 1000 rpm for 10 min. Later, the particles were washed in the order of cyclohexane once, water: ethanol: acetone mixture (1:1:1) two times, and with acetone once by repeated centrifugation. The particles were dried in vacuum oven for further use.

The p(Gly) micro particles were imaged with an optic microscope (Olympus, BX53, Tokyo, Japan), and scanning electron microscopy (SEM, Hitachi Ultra High-Resolution Analytical FE-SEM SU-70, Tokyo, Japan). The SEM images of p(Gly) particles were acquired at an operating voltage of 20 kV after placing the particles onto carbon tape-attached aluminum SEM stubs, and coating with gold to a thickness of a few nanometers in vacuum. The thermal characterization of p(Gly) particles was carried out using a thermogravimetric analyzer (SII TG/DTA 6300, Tokyo, Japan). About 4 mg of p(Gly) particles was placed in a ceramic pan, and the weight lost was documented in the temperature range of 50–500 °C (heating rate: 10 °C/min under a dry N_2_ flow with a rate of 100 mL min^−1^). The functional group analysis of Gly and p(Gly) particles was assessed using FT-IR spectroscopy (Nicolet IS10, Thermo, Waltham, MA, USA), by taking the corresponding FT-IR spectra of the materials in the spectral range of 4000–650 cm^−1^ with a resolution of 4 cm^−1^, using the ATR technique. The average size of the synthesized p(Gly) particles was measured using SEM images of the particles with the software ImageJ. The surface charge of p(Gly) particles at different pHs was determined by using Zetapals zeta potential (90 plus, Brookhaven Instrument Corp., Holtsville, NY, USA) analyzer. Potentiometric titration of p(Gly) particles was carried out in a 100-mL beaker for the determination of acid–base characteristics. For this purpose, 50 mg of p(Gly) particles were placed into 50 mL 10 mM KNO_3_ solution, and the titrations were carried out using 0.01 M HCl and NaOH as titrants. The potentiometric titrations were performed in the pH range of 1.5–12.

### 2.3. Blood Compatibility Studies of P(Gly) Particles

#### 2.3.1. Hemolysis Assay of P(Gly) Particles 

Blood compatibility tests of prepared p(Gly) particles were carried out by hemolysis and blood clotting tests in accord with the literature [29]. Hemocompatibility tests for p(Gly) particles were performed in accordance with a procedure obtained by the approval of Human Research Ethics Committee of Canakkale Onsekiz Mart University (2011-KAEK-27/2022). The human blood was freshly taken from volunteers and directly placed into anticoagulant (ethylenediamine tetraacetic acid, EDTA) containing sterile tubes. First, p(Gly) particles were sterilized under UV light exposure at 420 nm for 3 min. In the hemolysis analysis, 10, 5, 2.5, and 1 mg of p(Gly) particles were placed in tubes, then diluted in 10 mL of 0.9% saline solution and incubated at 37 °C in a shaking water bath. Then, 2 mL of EDTA-containing blood was diluted with 2.5 mL of 0.9% saline solution, and 0.2 mL of diluted blood solution was added to 10 mL of p(Gly) particle suspension and incubated at 37.5 °C for 1 h in a shaker bath. After this period, the suspensions were centrifuged at 100 g for 5 min and the absorbance of the supernatant solutions was measured by UV–vis spectrophotometer at 542 nm to determine the released amount of hemoglobin. The hemolysis% ratio was calculated using Equation (1).

(1) $$Hemolysis\;ratio\%=\frac{\left(Asample-Anegative\right)}{\left(Apositive-Anegative\right)}\times 100$$

where *A_sample_* is the absorbance of sample-containing blood solution, *A_positive_*, and *A_negative_* 0.2 mL diluted blood in 10 mL DI water, and 0.2 mL diluted blood in 10 mL saline solution without sample, respectively. Hemolysis tests were carried out three times, and data are given as the average of these values with standard deviations.

#### 2.3.2. Blood Clotting Assay of P(Gly) Particles 

In the blood clotting analysis, 10, 5, 2.5, 1 mg of p(Gly) particles were placed into sterile tubes. Particle containing tubes were kept in a water bath for 10–15 min until they reached 37 °C. In another tube, 810 µL of the fresh blood was interacted with 64 µL of 0.2 M calcium chloride (CaCl_2_) solution and 270 µL of this solution was placed on the p(Gly) particle containing tubes. These tubes were incubated at 37 °C for 10 min. Then, 10 mL of DI water was slowly added into the tubes and centrifuged at 100 g for 1 min. Next, 40 mL of DI water added into the supernatant solutions to dilute and the absorbance value at 542 nm was measured by UV-Vis spectrophotometer (T80+ PG Instrument). Blood clotting index of the p(Gly) particles was calculated employing Equation (2).

(2) $$Blood\;clotting\;index=(A_{\mathrm{SAMPLE}}/A_{\mathrm{BLOOD}})\times 100$$

where *A*_SAMPLE_ is the absorbance of the blood solution that interacted with sample, while *A*_BLOOD_ is absorbance of the 0.25 mL blood in 50 mL DI water. The test was repeated three times, and the average values with standard deviations are presented.

### 2.4. Cell Viability Assay of P(Gly) ) and QC Loaded P(Gly) Particles 

The cytotoxicity analyses of p(Gly) and Q@p(Gly) particles were performed on L929 fibroblast cells according to the literature [30]. Briefly, the live cells containing 1 × 10^4^ cells were placed in a 96-well plate and incubated for 24 h in a 5% CO_2_/95% air atmosphere at 37 °C. At the end of 24 h, the cells were checked, and the media was removed. P(Gly) particles and QC loaded p(Gly) particles were sterilized via UV irradiation (λ = 420 nm) for 3 min and these particles were suspended in DMEM growth medium to obtain 50, 100, 250, 500, and 1000 μg/mL concentration samples. Then, these samples were added to the 96-well plate and was kept in a CO_2_ incubator (5% CO_2_/95% air atmosphere) for 24 h, 37 °C. After this period, the old culture media (containing samples) were discarded, and the wells were washed with PBS twice. Then, 0.1 mL of 0.5 mg/mL MTT solution was added into wells, and the well-plate was incubated at 37 °C for 2 h in the dark. After this period, the MTT solution was removed and 0.2 mL of DMSO was added to dissolve formazan crystals, which were produced by living cells. Cell viability percentage was measured with an Elisa Microplate Reader (Thermo Scientific, Multiskan GO, Waltham, MA, USA) at 590 nm wavelength. The tests were performed in triplicate.

### 2.5. Quercetin (QC) Loading and Release Studies from P(Gly) Particles 

QC was loaded into p(Gly) particles as a therapeutic agent through the adsorption technique. Briefly, 10 mg of QC was dissolved in 30 mL of ethanol-water solution (at 1:1 by volume), and 50 mg of p(Gly) was put into an QC-solution-containing beaker. The particle suspension was stirred continuously for four hours at 200 rpm during the loading process. Afterward, the particles were centrifuged at 10,000 rpm for 10 min and dried in an oven. The amount of QC-loaded was determined by the absorbance difference of the QC solution via UV-vis spectroscopy by the corresponding pre-generated calibration curves at 307 nm for the QC solution prepared in the ethanol: water mixture.

A 10 mg of QC-loaded p(Gly) particle was placed into a dialysis membrane after dispersing in 2.5 mL of PBS. In a shaker bath at 37 °C, 27.5 mL of PBS solution was added to this particle-containing membrane. The released QC amount was measured against previously constructed calibration curves that were prepared in PBS by a UV-vis spectrometer at 376 nm, and the QC release amounts were calculated. Analyses were repeated three times, and averages and standard deviations were reported.

### 2.6. Antioxidant Studies of QC Loaded P(Gly) Particles

Antioxidant tests, total phenol content (TPC) tests, and total flavonoid tests (TFT) were performed according to the literature with some modifications [31]. Briefly, 2.0 mg/mL of QC@p(Gly) particles were prepared and mixed at 500 rpm overnight. This suspended solution was diluted as 1000, 500, 250, 125 µg/mL. An amount of 20 µL was put to into a 96 well plate, and 125 µL 0.2 N FC solution was added to this sample solution. Then, 100 µL of 0.7 M Na_2_CO_3_ aqueous solution was added to the medium and kept for two hours in the dark. Then, the solutions were read with a microplate reader at 760 nm (Thermo Scientific, Multiskan GO, Waltham, MA, USA). The calibration curve was prepared with gallic acid (GA), and the values were given as GA equivalent.

For the total flavonoid content (TPC), 50 µL of QC@p(Gly) suspended solution was put into 96 well. Then, 25 µL 3% NaNO_2_ was put into the solution. Then, 6% AlCl_2_ solution was put into the solution and finally, 100 µL of 1 M NaOH solution was added. After 15 min, the solutions were read at 405 nm against a previously generated calibration of TPC values of QC standard, and the findings were provided as µg/mL QC equivalency of total phenol content.

## 3. Results and Discussion

**Figure 1:**
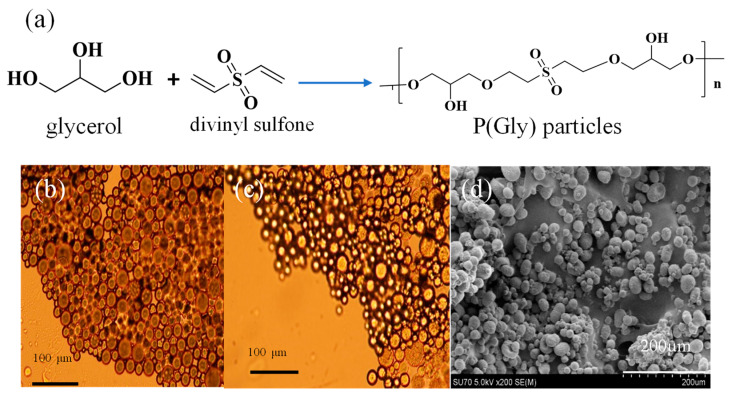
(**a**) The schematic presentation of p(Gly) particle preparation and the microscope image of (**b**) p(Gly) particle, (**c**) water swollen p(Gly) particles, and (**d**) SEM image of p(Gly) particles.

Microparticles of Gly were synthesized by the crosslinking of Gly with DVS crosslinker at 75% mol ratio of Gly via the microemulsion method in one step. The schematic representation of the p(Gly) particles is shown in Figure 1a. The microscope images of dry and swollen p(Gly) particles are shown in Figure 1b,c, respectively. It is realized from Figure 1b,c that the microparticles are highly water swollen. As can be seen in Figure 1d, the particles are a spherical shape. With the help of SEM images and ImageJ program, the average size of the particles was determined to be. 14.5 ± 5.6 µm.

Five different cross-linker ratios were tested in p(Gly) particle synthesis. The particle formation could only be achieved at three cross-linker ratios of 100, 75, and 50%. The amounts of particles and/or co-polymeric structures obtained at 37.5% and 18.8% cross-linker ratios was too low to be calculated. As the particle yield is negligible or nonexistent at ≤37.5% cross-linker ratio, it is presumed that critical cross-linking is >37.5 and approximately 50% at these three cross-linker ratios. The highest amounts of gravimetric yield were obtained p(Gly) particles prepared at 75%. The reason for not having higher gravimetric yield for 100% cross-linker ratio could be ineffective and self-crosslinking of DVS for p(Gly) particles formation. In Table 1, the parameters, such as gravimetric yield and zeta potential values at different solution pH, are given depending on the used amounts cross-linkers. The continuation of the study was carried out on particles with a high yield of 75% cross-linker. As shown in Table 1, the highest yield was determined as 58.1 with 75% crosslinker. Moreover, the zeta potential of the same particles in the zeta 10 mM KNO_3_ fraction was 47.4 and the pH was 5.9. 

The gravimetric yield of p(Gly) particles prepared at 100% and 50% cross-linker ratios were determined as 33.9 ± 2.1% and 37.2 ± 0.5%, respectively. The zeta potential value of 100% cross-linked p(gly) particles was measured as −63.1 ± 2.7 mV, whereas the 50% cross-linked p(Gly) particles had a zeta potential value of −28.7 ± 3.0 mV. As the cross-linker ratio increases, the magnitude of negative zeta potential values of the particles increases. As expected, the loosely cross-linked particles’ mobility as well as their charge values under an electric field experienced by the particle are reduced.

**Figure 2:**
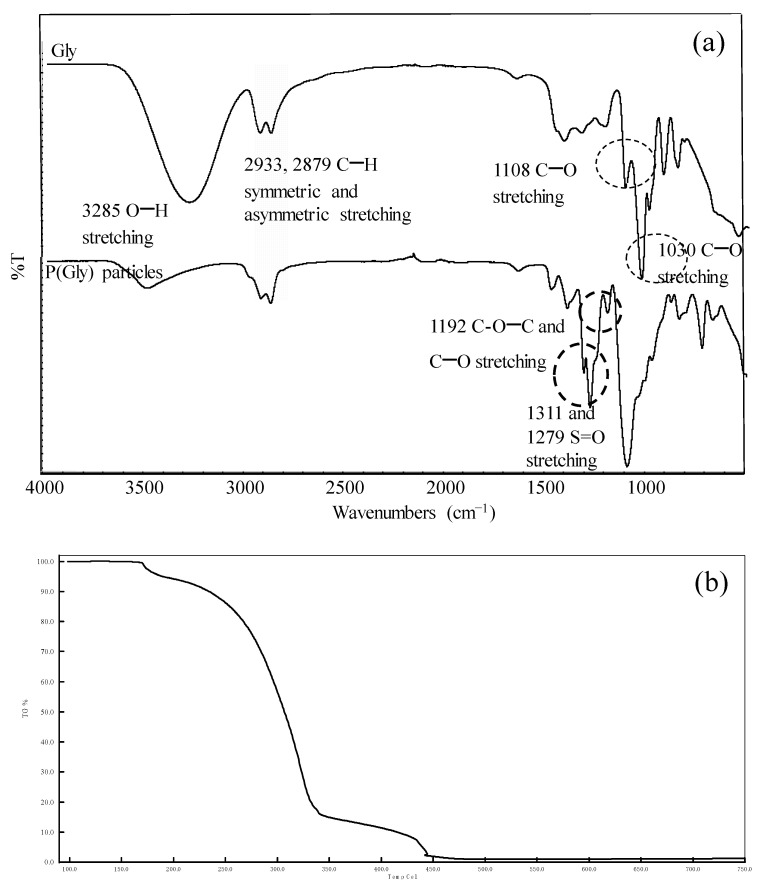
(**a**) FT-IR spectra of Gly and p(Gly) particles (75x), and (**b**) TG thermograms of p(Gly) particles.

From the FT-IR spectra of Gly molecule as shown in Figure 2a, the intensity of the broad band in 3200–3400 cm^−1^ ranges are due to the stretching vibration of the -OH groups. This peak was shifted for p(Gly) particles and the intensity of the peak decreased. The peaks at 2933 and 2879 cm^−1^ wavenumbers are the other evidence of -CH_2_ symmetric and asymmetric stretching bands of Gly. The C-O stretching peaks of Gly can be readily noticed in 1108 and 1030 cm^−1^ wavelengths. The peaks for S=O bonding are clearly detected at 1311 and 1279 cm^−1^. The FT-IR spectra analysis of p(Gly) particles provides evidence that cross-linking with DVS in the formation of p(Gly) particles was successful. The results of energy dispersive X-ray analysis (EDX) also confirm that Gly was crosslinked with DVS, as shown in Figure S1, due the presence of S atoms in p(Gly) particles.

Thermogravimetric analysis of p(Gly) particles was carried out using a thermogravimetric analyzer (TGA). The temperature-dependent weight loss of p(Gly) particles is given in Figure 2b. P(Gly) particles have four degradation stages in the temperature ranges in (1) 170–185 °C, (2) 220–340 °C, (3) 430–450 °C, and (4) up to 750 °C with 4.3%, 82.8%, 98.1%, and 98.8% weight losses, respectively, as illustrated in Figure 2b. 

**Figure 3:**
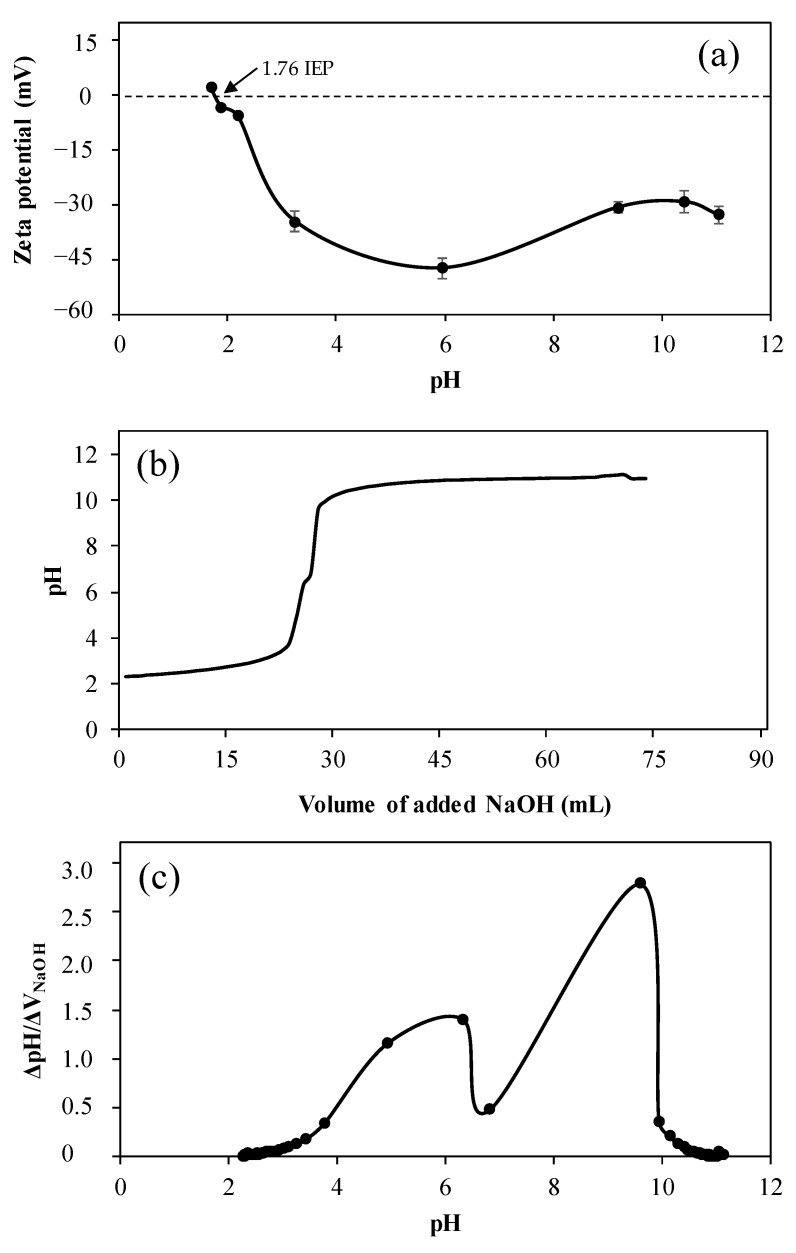
(**a**) Zeta potential measurements of p(Gly) particles at various pHs in 0.01 M KNO_3_ solutions, (**b**) potentiometric titration of p(Gly) particles, and (**c**) the equivalence points of the reaction calculated from the derivative of the potentiometric titration.

Zeta potential values of p(Gly) particles were measured in different pH solutions to determine the isoelectric point, and the results are shown in Figure 3a. According to the results, p(Gly) particles had a positive zeta potential value with +2.01 ± 3.2 mV at pH 1.69. The isoelectric point (IEP) of p(Gly) particles was found to be pH 1.76 where the zero-zeta potential value was measured and there is a balance between positive and negative charges on the particle surface at the IEP. Furthermore, potentiometric titration of p(Gly) particles was carried out in the pH range of 2–12 to calculate pKa values of the p(Gly) particles, as demonstrated in Figure 3b.

The potentiometric titration of p(Gly) particles was performed in 0.01 M 50 mL KNO_3_ solution using 0.1 M NaOH and 0.1 M HCl. It was found p(Gly) particles have two pKa value at pH 6.34 and 9.6, from hydroxyl groups, as shown in Figure 3c. This is reasonable as Gly molecule has three hydroxyl groups, one of which is completely employed in the crosslinking reaction with DVS.

**Figure 4:**
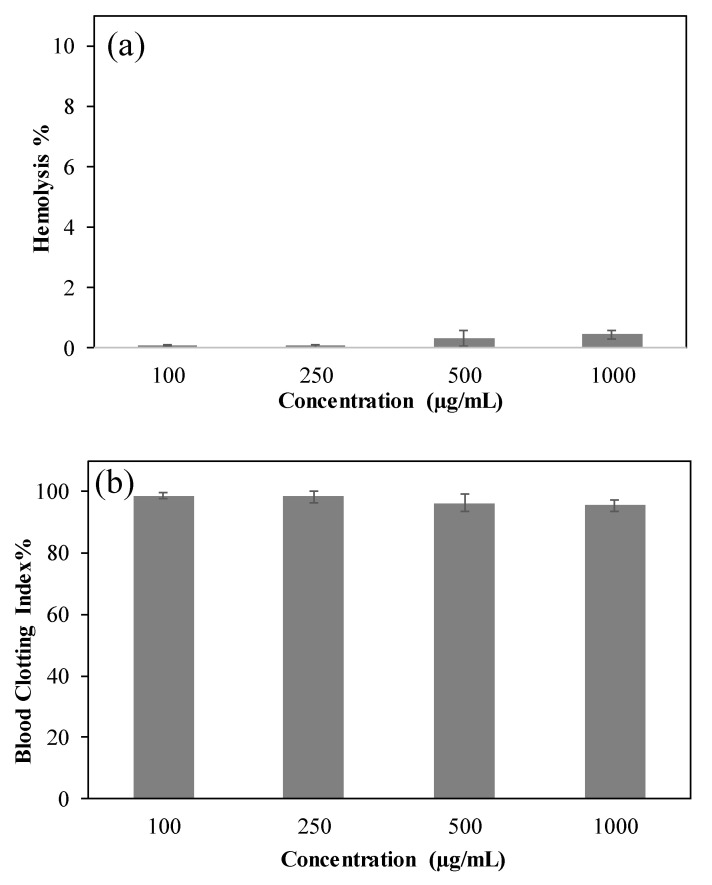
(**a**) hemolysis, and (**b**) Blood Clotting Index of p(Gly) particles at different concentration (100–1000 µg/mL).

The hemocompatibility of p(Gly) particles was determined by hemolysis and blood clotting index assays. Hemolysis% shows the blood toxicity of the materials on red blood cells and indicates possible usability of the materials for intravenous (IV) administration and/or blood contacting application, and the value of this should be at maximum 5% hemolysis to be considered as blood compatible [5]. The hemolysis% of the Glycerol particles at 100, 250, 500, 1000 µg/mL concentrations were found as 0.09 ± 0.01%, 0.07 ± 0.03%, 0.3 ± 0.2%, and 0.4 ± 0.1%, respectively, as shown in Figure 4a. These results indicated that Glycerol particles are non-hemolytic materials and could be used in blood contacting e.g., IV administration safely. On the other hand, the blood clotting test based on the clotting mechanism of blood interacting materials also needs to be considered for safe use. As illustrated in Figure 4b, the blood clotting index% values of Glycerol particles at 100, 250, 500, and 1000 µg/mL concentrations were determined as 98.5 ± 1.04%, 98.12 ± 1.9%, 96.07 ± 2.8%, and 95.4 ± 1.96%, respectively. A higher blood clotting index% value indicates higher hemocompatibility of the tested biomaterials [32]. Therefore, it can be confirmed that p(Gly) particles are blood compatible materials with blood clotting indexes% above 95%, which expressed no significant effects on the blood clotting mechanism. Hence, these p(Gly) particles could be used as scaffolds for wounds in certain injuries that require blood contact. Due to their hemocompatibility features, the prepared p(Gly) particles are promising biomaterials in blood contacting applications.

**Figure 5:**
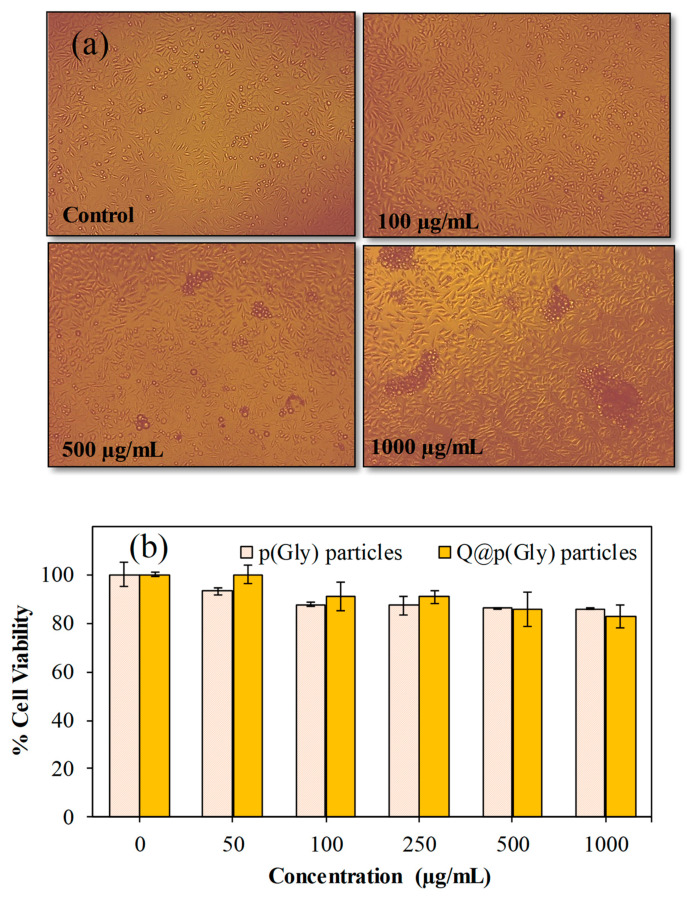
(**a**) Optic microscope images of L929 fibroblasts as control groups and p(Gly) particles at 100, 500 and 1000 µg/mL concentrations, and (**b**) Cytotoxicity of p(Gly) and Q@p(Gly) particles on L929 fibroblasts at 24 h incubation time.

The cytotoxicity levels of p(Gly) and Q@p(Gly) particles at different concentrations ranging from 50 to 1000 µg/mL were determined on L929 fibroblast cells for a 24-h incubation time, and the results are summarized in Figure 5.

Cell viability percentages of the fibroblasts in the presence of p(Gly) and Q@p(Gly) particles even at 1000 µg/mL concentration were found as 85 ± 1% and 82 ± 4%, respectively, showing that p(Gly) particles are biocompatible against fibroblasts and could be used for further in vivo applications up to 1000 µg/mL concentrations.

**Figure 6:**
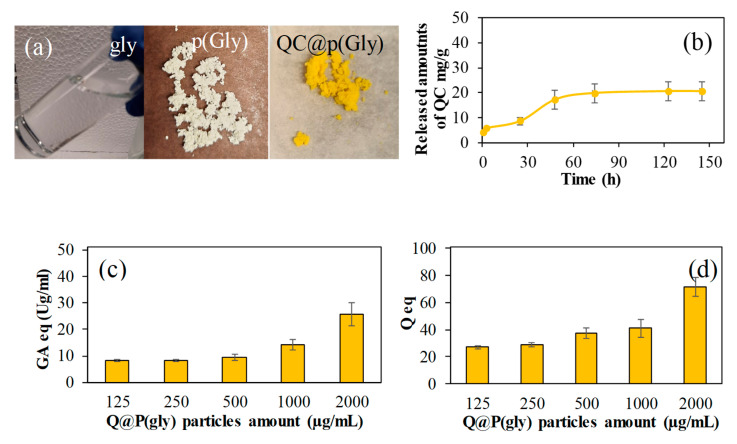
(**a**) Photograph images of Glycerol, p(Gly) and Q@p(Gly) particles, (**b**) in vitro drug release of Q@p(Gly) particles in PBS at pH 7.4, (**c**) Total phenol GA equivalency of Q@p(Gly) particles at different concentrations, and (**d**) Total flavonoid equivalency (Q eq) for Q@p(Gly) particles at different concentrations.

In the literature, loading of polyphenols into particles as a therapeutic agent is quite common. For example, curcumin as a hydrophobic polyphenol was loaded to the gelatin microgel for synergistic therapy of colorectal cancer [20]. In this study, QC is loaded into the particles via adsorption process as described earlier. The amount of QC loaded into p(Gly) particles is 380 ± 80 mg/g. As can be seen from Figure 6a, QC was loaded into p(Gly) particles, resulting in a color change from white color for bare p(Gly) to yellow color upon QC loading. Figure 6b shows the QC release profile from Q@p(Gly) particles at pH 7.4 and 37 °C. The QC release mechanism from QC loaded p(Gly) was assumed to be diffusion-controlled drug release as the drug is first dispersed throughout the p(Gly) microparticles and then diffuses through the polymeric network into the solution within the dialysis membrane. As seen, the release of QC from the particles is linear up to 50 h, and then the release rate sharply decreases between 50 h and 74 h with the total QC released amount of 20 mg/g. The released amount of QC is very little in comparison to the uploaded amount of QC. Quercetin is not a drug molecule on its own, but QC loaded p(Gly) particles can be used as a complementary therapeutic agent. For example, the QC loaded p(Gly) particles can be used as wound dressing/wound healing material. A biocompatible material, such as QC loaded p(Gly), can prevent skin from potentially harmful radicals upon contact with the skin because of the quite promising antioxidant properties of QC within the first 50 h. In addition, the release profile that can be described as sustained (lasting 75 h) is important in terms of the role of complementary therapy in therapeutic treatment. As QC is a hydrophobic molecule, and sparingly soluble in the PBS environment, it can be released for an extended time. Additionally, there could be hydrogen bond formation between the QC molecule and p(Gly) particles. In the QC release studies at the end of 150 h, the p(Gly) particles still possessed a yellow appearance within the membrane, indicating the high amounts of the presence of QC. Thus, it can be expected that there will be a longer time QC release in a linear fashion in an in vivo environment.

The total phenol potency of Q@p(Gly) particles in terms of GA equivalency is illustrated in Figure 6c. It is clear from the Figure 3c that the increase in amounts of the Qp(Glys) particles lead to an increase in antioxidant potency carrier systems in terms of GA equivalency, as expected. This is reasonable as the higher amounts of QC in the higher amounts, i.e., 2000 µg/mL, of Q@p(Gly) particles showed 25.7 ± 4.3 GA eq (µg/mL). Similar results were obtained for the total flavonoid test, as demonstrated in Figure 6d. In Figure 6d, the total flavonoid test results revealed the similar results, e.g., 2000 mg/mL of Q@p(Gly) particles, which is the highest amount studied, showed the maximum QC equivalency, 71.8 ± 7.1 QC eq (µg/mL). Therefore, it can be assumed that p(Gly) particles can be used as drug delivery materials including antioxidant phenolic compounds, in addition to antibiotic, antifungal, and anticancer drugs.

## 4. Conclusions

In this study, p(Gly) particles were synthesized using DVS as cross-linker via a micro emulsion technique for the first time. The particle synthesis was affirmed via SEM and FT-IR spectroscopy analyses. The p(Gly) particles were found to be spherical in shape within the size range of 10–20 µm. P(Gly) particles showed excellent blood compatibility with >95% blood clotting index values, even at 1 mg/mL concentrations, and were found to be non-hemolytic with <1% hemolysis ratios at the same concentration. Cytotoxicity studies of p(Gly) particles performed on L929 fibroblasts also revealed that these particles are biocompatible with no toxicity up to 1 mg/mL concentrations. Additionally, QC as potential therapeutic agent was successfully loaded into p(Gly) particles and the loaded particles demonstrated a capable drug loading and release capacity. P(Gly) microparticles can be injectable biomaterials for suitable drug carrying vehicles with high biocompatibility, blood compatibility, and non-toxicity with an average diameter of 15 µm. By loading p(Gly) microparticles with a wide variety of therapeutic agents, such as polyphenolics, antibiotics, antifungals, anticancer etc., these particles can be used as non-allergic wound dressing materials. Moreover, p(Gly) microparticles can provide an important field in the treatment of infections and chronic wounds caused by resistant bacteria by improving their drug loading and release capacities, and they are also highly promising for in vivo applications with the advantage of superior biocompatibility. In fact, our future studies will focus on some in vivo use of p(Gly) particles.

## Figures and Tables

**Table 1:** Gravimetric yields, zeta potentials, pH and mobility of p(Gly) particles.

Used Crosslinker Ratio (mole%)	Gravimetric Yield	Zeta Potential (mV)	Solution pH	Mobility (µ/s)/(v/cm)
100	33.9 ± 2.1	−63.1 ± 2.7	5.51	−4.9 ± 0.2
75	58.1 ± 2.0	−47.4 ± 2.9	5.96	−3.8 ± 0.2
50	37.2 ± 0.5	−28.7 ± 3.0	9.19	−2.2 ± 0.2
37.5	-			
18.8	-			

## Data Availability

The data presented in this study are available on request from the corresponding author.

## Supplementary Materials

The following supporting information can be downloaded at: https://www.mdpi.com/article/10.3390/pharmaceutics15020384/s1, Figure S1: Energy-dispersive X-ray spectroscopy analysis of p(Gly) particles.
