# Supplementary figures and images for: Poly(Glycerol) Microparticles as Drug Delivery Vehicle for Biomedical Use
Source: Pharmaceutics. 2023 Jan 23;15(2):384. doi: 10.3390/pharmaceutics15020384 (PMC9964732; doi:10.3390/pharmaceutics15020384)

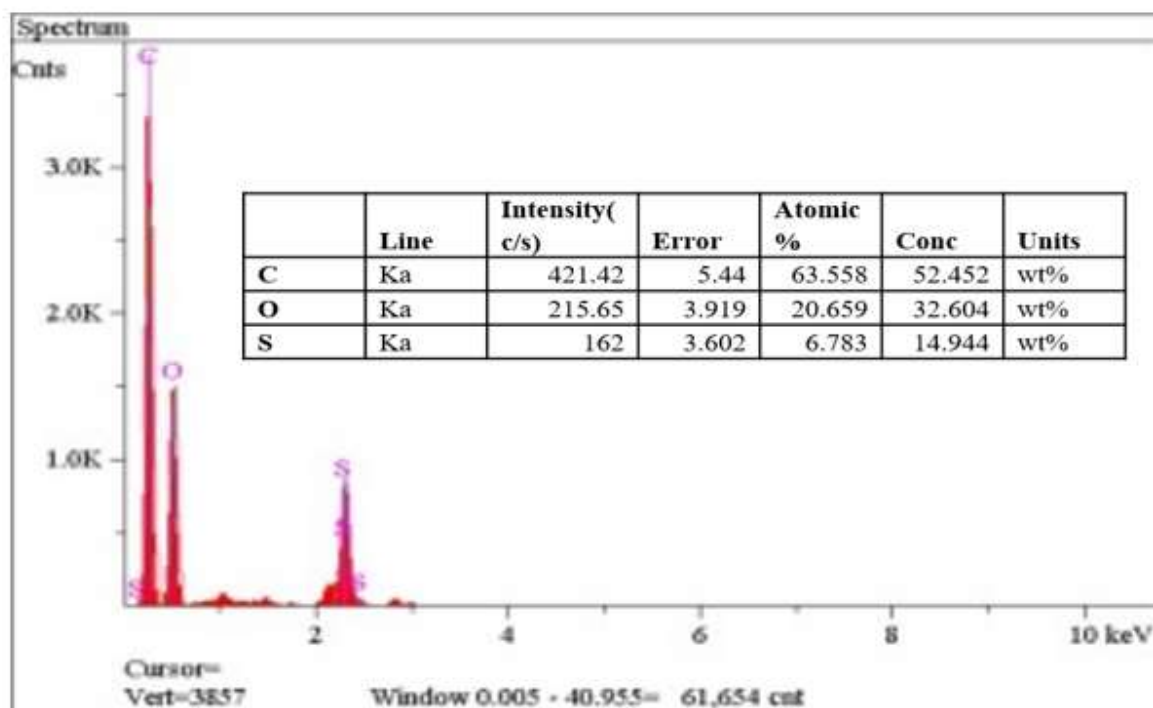

**Figure S1.** Energy-dispersive X-ray spectroscopy analysis of p(Gly) particles.

Supplement: Supplementary file 1 [file pharmaceutics-15-00384-s001.zip › pharmaceutics-2136753-supplementary.pdf]
